# STAT3 signaling pathway plays importantly genetic and functional roles in HCV infection

**DOI:** 10.1002/mgg3.821

**Published:** 2019-06-20

**Authors:** Yuzhu Song, Xianyao Yang, Yunsong Shen, Yiqian Wang, Xueshan Xia, A‐Mei Zhang

**Affiliations:** ^1^ Faculty of Life Science and Technology Kunming University of Science and Technology Kunming China; ^2^ Molecular Medicine Center of Yunnan Province Kunming China

**Keywords:** biochemical characteristics, functional, genetic, HCV, STAT3 signaling pathway

## Abstract

**Background:**

Hepatitis C virus (HCV) infection is an extensive health problem, which leads to serious liver diseases. Host genetic polymorphisms were associated with HCV infection, progression, and treatment effect of patients. Signal transducers and activators of transcription 3 (STAT3) signaling pathway was important to HCV infection, but no genetic association was studied between STAT3 signaling pathway and HCV infection.

**Methods:**

To investigate the genetic and functional roles of the STAT3 signaling pathway, we collected 394 HCV patients and 395 normal controls to genotype 25 signal nucleotide polymorphisms (SNPs) of six genes (Interleukin 6 [*IL6,* OMIM 147620], Interleukin 6 receptor [*IL6R*, OMIM 147880], Hepatocyte nuclear factor 1 alpha [*HNF1A*, OMIM 142410], Hepatocyte nuclear factor 4 alpha [*HNF4A*, OMIM 600281], *STAT3* [OMIM 102582], and ATP binding cassette subfamily C member 2 [*ABCC2*, OMIM 601107]). Then expression level of these genes were analyzed in HCV infected cells with or without IL6 transfection.

**Results:**

Our results identified that the SNPs in STAT3 signaling pathway were associated with HCV infection or biochemical features of Yunnan HCV patients. Genotype AA of rs4075015 (*IL6R*) and GG of rs3212172 (*HNF4A*) increased the risk of HCV infection (*p* = 0.024 and 0.029), but the genotype AA of rs7553796 (*IL6R*) played a protective role in HCV infection (*p* = 0.0008). High‐density lipoprotein cholesterol (HDL‐C) and Glutamyl transpeptidase (GGT) level were associated with genotypes of rs4845617 (*IL6R*, *p* = 0.045) and rs1053023 (*STAT3*, *p* = 0.034), respectively. Cell assays suggested that IL6 transfection could suppress HCV proliferation. RNA and protein levels of the *IL6R*, *HNF1A*, *STAT3*, and *ABCC2* genes significantly increased after HCV infection.

**Conclusion:**

We identified STAT3 signaling pathway influenced HCV infection and biochemical characteristics of HCV patients through genetic and functional aspects.

## 1 INTRODUCTION

Hepatitis C virus (HCV) infection is one of causative reasons of chronic hepatitis and serious liver diseases worldwide. About 180 million persons are infected by HCV in the world (Micallef, Kaldor, & Dore, 2006). Although direct‐acting antivirals (DAAs) greatly increase curative ratio of HCV infected persons, wide prevalence, high mutant rate, presence of cirrhosis, and expansive cost bring to difficulties for HCV therapy (Bagaglio, Uberti‐Foppa, & Morsica, 2017; Hayes & Chayama, 2017). Thus, it is important to further understand the pathogenic mechanisms of HCV infection and disease progression. Host genetic susceptibility is associated with HCV infection, viral clearance, and therapy effect, and most of these susceptibility genes belong to interleukin family (Falleti et al., 2010; Kao et al., 2017; Sghaier et al., 2017), which is important cytokine in immunoreaction.

Genetic polymorphisms in the interleukin 28B gene (*IL28B*, OMIM 607402) are widely identified to associate with viral clearance and therapy effect in many populations (Ge et al., 2009; Thomas et al., 2009). At the same time, chronic HCV infection could decrease the IL28B serum level (Alborzi et al., 2017). Similarly, the interleukin 6 gene (*IL6*) plays an important role in human immunologic antagonism, so the genetic variations in the *IL6* gene are commonly studied in HCV infected persons (Cussigh et al., 2011). Single nucleotide polymorphism (SNP) of the *IL6* gene is associated with HCV viral clearance, and the serum IL6 level shows higher in HCV infected persons (Tarrago et al., 2014). All above, the genetic polymorphisms of the interleukin genes could influence the infection, pathopoiesis, and treatment effect of HCV patients. Until now, no study was performed to reveal the genetic role of a signaling pathway in HCV infected population.

The proteins in signal transducer and activator of transcription (STAT) family are identified to be critical factors in cytokine signaling pathway. Mutations in the *STAT3* gene could lead to many kinds of cancers (Shahmarvand, Nagy, Shahryari, & Ohgami, 2018). STAT3 protein together with IL6, HNF4A, HNF1A, and three microRNAs constructed a feedback loop, which regulates oncogenesis of hepatocellular carcinoma (HCC) (Hatziapostolou et al., 2011). HNF4A is essential for liver function and regulates the expression level of HNF1A, which controls several genes of hepatic development. IFN signaling and IL6 signaling pathways could activate STAT3 protein when HCV core protein highly expressed (Tacke, Tosello‐Trampont, Nguyen, Mullins, & Hahn, 2011). In addition, the *ABCC2* gene encodes a member in the ATP‐binding cassette (ABC) transporters superfamily, which expression level decreased in HCC tissues induced by HCV (Billington et al., 2018). Interestingly, the *ABCC2* gene locates in downstream of the HNF1A and HNF4A genes (Qadri, Iwahashi, Kullak‐Ublick, & Simon, 2006). Thus, the IL6/HNF4A/HNF1A/STAT3/ABCC2 signaling pathway might participate in HCV infection, pathogenic process, and treatment effect, but whether genetic variations of these genes could influence HCV infection remains unclear.

In this study, we investigated whether there was relationship between genetic polymorphisms of genes in STAT3 signaling pathway and HCV infection in Yunnan population, and validated the function of STAT3 pathway in HCV‐infected cells.

## 2 MATERIALS AND METHODS

### 2.1 Ethical complicance

Written informed consents conforming to the tenets of the Declaration of Helsinki were obtained from each participant prior to the study. The institutional review board of Kunming University of Science and Technology approved this study (Approval No. 2014SK027).

### 2.2 Subjects

394 chronic HCV‐infected subjects and 395 general controls were recruited in First People's hospital of Yunnan Province. The patients were diagnosed as chronic HCV infected persons by the symptoms and liver function test. All HCV‐infected patients were identified to be anti‐HCV positive by HCV ELISA Kit (ORTHO, USA), and all patients were without any medical treatment when we collected the samples. None individual carried serious liver disease, and all individuals were without Hepatitis B virus (HBV) infection detected by using Quantitative CLIA Kit (Autobio, China)and/or Human Immunodeficiency virus (HIV) infection detected by using Anti‐HIV ELISA Kit (WANTAI, China). The persons, who were anti‐HCV positive, HBV DNA negative, and anti‐HIV negative, were classified to HCV‐infected patients group. Moreover, controls were anti‐HCV, HBV DNA, and anti‐HIV negative. The basic information and biochemical characteristics [including Glutamyl transpeptidase (GGT) glutamic‐pyruvic transaminase (GPT or ALT), aspartate amino‐transferase (GOT or AST), albumin (ALB), total bilirubin (TBIL), and high‐density lipoprotein cholesterol (HDL‐C)] of all subjects were collected for further analysis. 5 ml whole blood were collected from each subject for single nucleotide polymorphism (SNP) analysis.

### 2.3 Genomic DNA extraction and genotyping

Genomic DNA was extracted from whole blood by using TIANamp Blood DNA Kit (TIANGEN, China). Twenty‐five SNPs (rs1524107, rs2069837, rs2069840, rs2069852, rs4845617, rs12090237, rs4075015, rs7553796, rs4845374, rs4509570, rs1053023, rs1053004, rs4796793, rs3787349, rs3212172, rs1800963, rs6073435, rs1169309, rs11065390, rs41279096, rs1885301, rs7910642, rs2804402, rs717620, and rs2273697) of six genes (*IL6*, *IL6R*, *HNF1A*, *HNF4A*, *STAT3*, and *ABCC2*) in STAT3 signaling pathway were selected according to the criteria that the minor allele frequency of SNP is more than 2% in dbSNP (www.ncbi.nlm.nih.gov/SNP). These SNPs were tagSNPs (ftp.ncbi.nlm.nih.gov/hapmap/, CHB + JPT) or SNPs in functional region (coding region, 5′‐untranslated region, and 3′‐untranslated region). Then, each SNP was genotyped by using Ligase Detection Reaction (LDR) or SnapShot assay (the primers were listed in Tables S1 and S2). The genotyping results of random 10% samples with each SNP were verified by sequencing. The linkage disequilibrium (LD) maps for SNPs in each gene were calculated by using SHEsis (analysis.bio‐x.cn/myAnalysis.php) (Shi & He, 2005) and listed in Figure S1.

### 2.4 HCV RNA extraction, genotyping and quantification

HCV RNA was abstracted from serum of each HCV‐infected individual by using TIANamp Virus RNA Kit (TIANGEN, China). Two pairs of primers (HCV‐1F/HCV‐1R: 5′‐ GGCGACACTCCACCATGAATCACT‐3′/5′‐ GTATGTACCCCATGAGGTCGGC‐3′; HCV‐2F/HCV‐2R: 5′‐ AATCACTCCCCTGTGAGGAACTACTGT‐3′/5′‐ AGGGTATCGATGACCTTACCCA‐3′) were designed to amplify NS5B gene of HCV by using nest PCR (Lu et al., 2005; Zhang et al., 2014). The PCR condition was as following: one cycle of 94°C for 5 min, 30 cycles of 94°C for 30 s, 50°C for 30 s, and 72°C for 50 s, and one extension cycle of 72°C for 5 min. The NS5B fragment was sequenced to genotype HCV. Taqman probe assay was used to detect HCV viral load by primers HCV‐3F/HCV‐3R (5′‐TGCTCATGATGCACGGTCTAC‐3′/5′‐TGCGGAACCGGTGAGTACAT‐3′), probe (FAM‐CACCCTATCAGGCAGTACCACAAGGCC‐TAMRA), and ABI 7,500 Fast Real‐Time PCR system (Applied Biosystems, USA). The lowest limit of detection was 1,000 copies/mL. HCV RNA was further transformed as log10‐transformation, i.e., the lowest limit of detection was 3 log10 copies/mL.

### 2.5 Plasmid construction

Total RNA was extracted from Huh7.5.1 cells by using TRIzol Reagent (Invitrogen, USA). 1 μg RNA was reversely transcripted to complementary DNA (cDNA) by using PrismScript^TM^ RT reagent Kit with gDNA Eraser (Takara, Japan). The *IL6* gene (GenBank No.: NM_000600) was amplified by using primers IL6‐F and IL6‐R: CCG*CTCGAG*ATGAACTCCTTCTCCACA/GGG*AAGCTT*CTA**CTTGTCATCGTCGTCCTTGTAATC**CATTTGCCGAAGAGC. The italic sequences in primers were for endonuclease, and the bold sequence was to encode flag tag. A total of 50 μL PCR reaction volume (containing 5 μL 10× PCR buffer, 1.25 unit of Pfu DNA Polymerase [Promega, USA], 200 μM of each dNTP, 0.5 μM of each primer, and 50 ng genomic DNA) was performed by using following PCR condition: one cycle of 95°C for 1 min, 30 cycles of 95°C for 30 s, 58°C for 30 s, and 72°C for 30 s, and one extension cycle of 72°C for 5 min. Then, the PCR product was cloned into pcDNA3.1 plasmid (with flag tag in the terminal of cloned *IL6* gene) by using restriction endonuclease sites *Xho* I and *Hind* III.

### 2.6 Cell culture, transfection, and HCV infection

Huh7.5.1 cells were cultured in Dulbecco's modified Eagle's medium (DMEM; HyClone, USA) supplemented with 10% FBS and antibiotics (100 u/mL penicillin and 100 μg/mL streptomycin). After the cells were grown to 70% confluence in 6 wells plate, mixture of 2 μg IL6‐plasmid DNA and 2 μL of X‐tremeGENE HP DNA Transfection Reagent (Roche, USA) was transfected. Cells without any transfection and cells transfected with empty pcDNA3.1 vector were used as the controls. Cells were harvested at 24, 48, and 72 hr after the transfection.

HCV J6/JFH1 (muhiplieity of infection [MOI] = 1) was used to infect Huh7.5.1 cells with or without the *IL6* gene transfection. The infected cells were harvested at 24, 48, and 72 hr. The cells without HCV infection was used as controls.

### 2.7 Total RNA extraction and quantification

Total RNA of control and HCV infected cells was extracted by using TRIzol Reagent (Invitrogen, USA) and reversely transcripted into complementary DNA (cDNA) by using PrimeScript™ II 1st Strand cDNA Synthesis Kit (Takara, Japan). The mRNA level of five genes were quantified by using primers (listed in Table S3), SYB® Premix ExTaqTM II kit (TaKaRa Biotechnology Co. Ltd. [Dalian, China]), and ABI 7500 Real‐Time PCR System (Life, USA). The single‐copy nuclear GAPDH gene was used as the reference gene.

### 2.8 Elisa and Western blot

The IL6 protein level in supernatant of HCV infected Huh7.5.1 cells was detected by using IL‐6 Human ELISA Kit (Invitrogen, USA) according to the manufacturer's instruction.

Western blot was performed to detect the protein expression level of IL6R, HNF1A, HNF4A, STAT3, pSTAT3, and ABCC2. Firstly, Huh7.5.1 cells were collected to extract total protein. Then about 20 μg protein was electrophoresed in 8–12% SDS polyacrylamide gel and was transferred to 0.45 µm polyvinylidene difluoride membrane (Life, USA) under 120 V for 2 hr. The membrane was blocked in 5% (w/v) nonfat milk for 2 hr at room temperature, then incubated with monoclonal antibodies (Abcam, China) overnight at 4°C. Finally, the membrane was incubated with secondary antibody for 1 hr and visualized by using Tanon 5500 automatic Chemiluminescent imaging analysis system (Tanon, China).

### 2.9 Data analysis

The Hardy–Weinberg equilibrium (HWE) was assessed for each SNP to identify the deviation. Pearson Chi‐square test (with Yates’ correction) was used to analyze genotype and allele frequencies of SNPs between HCV‐infected persons and controls. The odds ratio (OR) and 95% confidence intervals (95% CI) were calculated. The association between genotype of SNPs and biochemical characteristics, the RNA expressing level of each subgroups, and HCV viral load between different HCV genotypes were evaluated by using Analysis of Variance (ANOVA) or Student's *t* test (with welch's correction, two‐tailed). The homozygous genotypes less than 10 were clustered into heterozygous genotypes of SNPs for biochemical characteristics analysis. A *P*<0.05 was considered as statistical significance. When the *P*<0.0001, *p* < 0.0001 was used.

## 3 RESULTS

### 3.1 Basic and biochemical information analysis

The gender ratio (male: female) was similar between HCV‐infected group (1:2.2) and controls (1:1.9) (Table 1). The biochemical characteristics GGT, ALT, AST, and HDL‐C showed significant difference between HCV‐infected individuals and controls, but ALB and TBIL were similar between them (Table 1). When the HCV‐infected persons and controls were divided into male and female group, similar results with total individuals were obtained, i.e. both GGT (*p* < 0.0001) and ALT (*p* = 0.02 and <0.0001) were significantly higher in both male and female HCV‐infected persons than controls, and ALB and TBIL showed no difference. Interestingly, AST was higher in female HCV‐infected group than controls (*p* < 0.0001), but it was similar between male HCV‐infected group and controls (*p* = 0.057). HDL‐C level was significantly lower in male HCV‐infected individuals than controls (*p* = 0.024), but no difference was found between female HCV‐infected individuals and controls (*p* = 0.173).

**Table 1:** Comparison of biochemical characteristics between HCV‐infected persons and controls

Reference range	HCV‐infected persons (*n* = 394)	Controls (*n* = 395)	*P*‐value
Total			
Male	123	138	0.122
Female	271	257	
GGT (3–50 U/L)	56.63 ± 3.29	26.80 ± 2.12	<0.0001
ALT (0–40 U/L)	67.27 ± 9.18	29.29 ± 1.07	<0.0001
AST (0–40 U/L)	66.84 ± 9.99	32.79 ± 0.79	0.0007
ALB (35–51 g/L)	44.91 ± 0.22	44.65 ± 1.11	0.818
TBIL (3.4–17.1 µmol/L)	19.48 ± 2.59	17.88 ± 0.61	0.549
HDL‐C (0.7–2.0 mmol/L)	1.25 ± 0.02	1.33 ± 0.02	0.020
Male			
GGT (3–50 U/L)	69.22 ± 6.64	30.75 ± 2.79	<0.0001
ALT (0–40 U/L)	101.80 ± 28.50	34.67 ± 1.82	0.020
AST (0–40 U/L)	95.37 ± 31.57	34.64 ± 1.52	0.057
ALB (35–51 g/L)	44.68 ± 0.54	45.04 ± 3.05	0.907
TBIL (3.4–17.1 µmol/L)	23.93 ± 5.19	22.80 ± 1.25	0.833
HDL‐C (0.7–2.0 mmol/L)	1.18 ± 0.04	1.31 ± 0.04	0.024
Female			
GGT (3–50 U/L)	50.91 ± 3.66	24.68 ± 2.89	<0.0001
ALT (0–40 U/L)	51.62 ± 2.98	26.40 ± 1.29	<0.0001
AST (0–40 U/L)	53.90 ± 2.22	31.80 ± 0.89	<0.0001
ALB (35–51 g/L)	45.02 ± 0.21	44.45 ± 0.46	0.253
TBIL (3.4–17.1 µmol/L)	17.46 ± 2.94	15.23 0.59	0.460
HDL‐C (0.7–2.0 mmol/L)	1.29 ± 0.03	1.34 ± 0.03	0.173

Abbreviations: ALB, albumin; ALT (or GPT), glutamic‐pyruvic transaminase; AST (or GOT), aspartate amino‐transferase; GGT, Glutamyl transpeptidase; HDL‐C, high‐density lipoprotein cholesterol; TBIL, total bilirubin.

### 3.2 Association between SNPS in STAT3 signaling pathway and HCV infection

All SNPs were not deviated from HWE in both case and control populations (Table 2). Genotyping and allele frequency were compared between HCV‐infected persons and controls (Table 2). Genotype AA of SNP rs4075015, which belongs to the *IL6R* gene, showed significantly higher frequency in HCV‐infected persons (82/394 = 20.8%) than controls (58/395 = 14.7%) (*p* = 0.024), but other genotype and allele frequencies were similar in two cohorts. Frequencies of genotype AA and CC of rs7553796 were statistically lower (*p* = 0.0008) and higher (*p* < 0.0001) in HCV‐infected persons than controls respectively. Similarly, allele A and C of rs7553796 showed the congruent trends in HCV‐infected individuals, i.e., HCV‐infected persons were with lower frequency of allele A (*p* < 0.0001) and higher frequency of allele C (*p* < 0.0001). Genotype GG of rs3212172 in the *HNF4A* gene showed higher frequency in HCV‐infected persons (29/394 = 7.4%) than controls (15/395 = 3.8%) (*p* = 0.029).

**Table 2:** Genotype and allele frequency of genes in STAT3 signaling pathway in HCV‐infected persons and controls

SNP	HCV patients (*n* = 394)	Controls (*n* = 395)	*P*‐value	OR (95% CI)^a^
rs1524107 (*IL6*)	HWE^b^ *p* = 0.076	HWE *p* = 0.083		
Genotype				
CC	26	22	0.545	1.198 (0.667–2.152)
CT	149	141	0.537	1.096 (0.820–1.464)
TT	219	232	0.371	0.879 (0.663–1.166)
Allele				
C	201	185	0.334	1.120 (0.890–1.409)
T	587	605		
rs2069837 (*IL6*)	HWE *p* = 0.063	HWE *p* = 0.103		
Genotype				
AA	255	265	0.483	0.900 (0.670–1.208)
AG	126	115	0.382	1.145 (0.845–1.550)
GG	13	15	0.705	0.864 (0.406–1.842)
Allele				
A	636	645	0.635	0.941 (0.731–1.211)
G	152	145		
rs2069840 (*IL6*)	HWE *p* = 0.115	HWE *p* = 0.24		
Genotype				
CC	349	362	0.149	0.707 (0.441–1.134)
CG	45	33	0.149	1.414 (0.882–2.269)
GG	0	0	‐	‐
Allele				
C	743	757	0.160	0.720 (0.454–1.141)
G	45	33		
rs2069852 (*IL6*)	HWE *p* = 0.053	HWE *p* = 0.054		
Genotype				
AA	185	187	0.913	0.985 (0.744–1.302)
AG	161	173	0.404	0.887 (0.668–1.176)
GG	48	35	0.128	1.427 (0.901–2.261)
Allele				
A	531	547	0.429	0.918 (0.742–1.135)
G	257	243		
rs4845617 (*IL6R*)	HWE *p* = 0.069	HWE *p* = 0.068		
Genotype				
AA	87	87	0.985	1.003 (0.717–1.405)
AG	182	193	0.453	0.899 (0.679–1.188)
GG	125	115	0.425	1.131 (0.835–1.533)
Allele				
A	356	367	0.610	0.950 (0.779–1.158)
G	432	423		
rs12090237 (*IL6R*)	HWE *p* = 0.306	HWE *p* = 0.182		
Genotype				
AA	1	2	0.564	0.500 (0.045–5.54)
AG	30	31	0.902	0.968 (0.574–1.632)
GG	363	362	0.802	1.067 (0.640–1.780)
Allele				
A	32	35	0.716	0.913 (0.559–1.490)
G	756	755		
rs4075015 (*IL6R*)	HWE *p* = 0.007	HWE *p* = 0.030		
Genotype				
AA	82	58	0.024	1.527 (1.055–2.211)
AT	160	199	0.902	1.017 (0.777–1.332)
TT	152	138	0.598	1.080 (0.810–1.443)
Allele				
A	324	315	0.615	1.053 (0.861–1.287)
T	464	475		
rs7553796 (*IL6R*)	HWE *p* = 0.074	HWE *p* = 0.004		
Genotype				
AA	66	105	0.0008	0.556 (0.393–0.786)
AC	189	216	0.059	0.764 (0.578–1.011)
CC	139	74	<0.0001	2.365 (1.706–3.278)
Allele				
A	321	426	<0.0001	0.587 (0.481–0.717)
C	467	364		
rs4845374 (*IL6R*)	HWE *p* = 0.025	HWE *p* = 0.090		
Genotype				
AA	7	6	0.776	1.173 (0.391–3.522)
AT	117	96	0.088	1.316 (0.960–1.804)
TT	270	293	0.079	0.758 (0.556–1.033)
Allele				
A	131	108	0.102	1.259 (0.955–1.66)
T	657	682		
rs4509570 (*IL6R*)	HWE *p* = 0.125	HWE *p* = 0.140		
Genotype				
CC	294	315	0.086	0.747 (0.535–1.043)
CG	91	74	0.132	1.303 (0.923–1.839)
GG	9	6	0.431	1.516 (0.534–4.300)
Allele				
C	679	704	0.075	0.761 (0.563–1.029)
G	109	86		
rs1053023 (*STAT3*)	HWE *p* = 0.007	HWE *p* = 0.033		
Genotype				
AA	175	166	0.498	1.102 (0.832–1.461)
AG	187	186	0.916	1.015 (0.768–1.342)
GG	32	43	0.186	0.724 (0.448–1.170)
Allele				
A	537	518	0.277	1.123 (0.911–1.386)
G	251	272		
rs1053004 (*STAT3*)	HWE *p* = 0.006	HWE *p* = 0.025		
Genotype				
CC	37	50	0.143	0.715 (0.456–1.122)
CT	192	195	0.858	0.975 (0.737–1.289)
TT	165	150	0.263	1.177 (0.885–1.565)
Allele				
C	266	295	0.137	0.855 (0.696–1.051)
T	522	495		
rs4796793 (*STAT3*)	HWE *p* = 0.015	HWE *p* = 0.015		
Genotype				
CC	159	147	0.365	1.141 (0.857–1.520)
CG	192	198	0.695	0.946 (0.715–1.250)
GG	43	50	0.447	0.845 (0.548–1.304)
Allele				
C	510	492	0.314	1.111 (0.905–1.364)
G	278	298		
rs3787349 (*HNF4A*)	HWE *p* = 0.016	HWE *p* = 0.051		
Genotype				
AA	16	19	0.609	0.838 (0.424–1.654)
AC	151	148	0.804	1.037 (0.778–1.383)
CC	227	228	0.976	0.996 (0.751–1.321)
Allele				
A	183	186	0.880	0.982 (0.778–1.240)
C	605	604		
rs3212172 (*HNF4A*)	HWE *p* = 0.064	HWE *p* = 0.007		
Genotype				
AA	206	226	0.164	0.819 (0.619–1.085)
AG	159	154	0.695	1.059 (0.796–1.408)
GG	29	15	0.029	2.013 (1.062–3.816)
Allele				
A	571	606	0.053	0.799 (0.637–1.003)
G	217	184		
rs1800963 (*HNF4A*)	HWE *p* = 0.100	HWE *p* = 0.070		
Genotype				
AA	30	34	0.609	0.875 (0.524–1.460)
AC	187	171	0.239	1.183 (0.894–1.567)
CC	177	190	0.371	0.880 (0.665–1.164)
Allele				
A	274	239	0.148	1.168 (0.946–1.441)
C	541	551		
rs6073435 (*HNF4A*)	HWE *p* = 0.008	HWE *p* = 0.063		
Genotype				
AA	161	168	0.635	0.934 (0.703–1.239)
AT	194	182	0.374	1.135 (0.858–1.501)
TT	39	45	0.496	0.854 (0.543–1.345)
Allele				
A	516	518	0.971	0.996 (0.809–1.226)
T	272	272		
rs1169309 (*HNF1A*)	HWE *p* = 0.056	HWE *p* = 0.069		
Genotype				
GG	104	117	0.313	0.852 (0.624–1.163)
GT	198	185	0.337	1.147 (0.867–1.516)
TT	92	93	0.949	0.989 (0.712–1.375)
Allele				
G	406	419	0.547	0.941 (0.772–1.147)
T	382	371		
rs11065390(*HNF1A*)	HWE *p* = 0.060	HWE *p* = 0.071		
Genotype				
AA	26	39	0.094	0.645 (0.385–1.082)
AG	160	155	0.695	1.059 (0.796–1.408)
GG	208	201	0.592	1.079 (0.816–1.427)
Allele				
A	212	233	0.253	0.880 (0.706–1.096)
G	576	557		
rs41279096 (*HNF1A*)	HWE *p* = 0.789	HWE *p* = 0.739		
Genotype				
AA	0	0	1.000	‐
AG	3	4	0.707	0.750 (0.167–3.373)
GG	391	391	0.707	1.333 (0.296–5.997)
Allele				
A	3	4	0.707	0.751 (0.168–3.366)
G	785	786		
rs1885301 (*ABCC2*)	HWE *p* = 0.055	HWE *p* = 0.083		
Genotype				
AA	13	20	0.216	0.640 (0.314–1.305)
AG	132	117	0.241	1.197 (0.886–1.617)
GG	249	258	0.535	0.912 (0.681–1.220)
Allele				
A	158	157	0.930	1.011 (0.790–1.294)
G	630	633		
rs7910642 (*ABCC2*)	HWE *p* = 0.029	HWE *p* = 0.043		
Genotype				
AA	40	39	0.896	1.031 (0.648–1.642)
AG	187	179	0.546	1.090 (0.824–1.442)
GG	167	177	0.492	0.906 (0.684–1.201)
Allele				
A	267	257	0.569	1.063 (0.862–1.311)
G	521	533		
rs2804402 (*ABCC2*)	HWE *p* = 0.062	HWE *p* = 0.087		
Genotype				
AA	252	257	0.746	0.953 (0.712–1.276)
AG	129	118	0.385	1.143 (0.846–1.544)
GG	13	20	0.216	0.640 (0.314–1.305)
Allele				
A	633	632	0.869	1.021 (0.797–1.308)
G	155	158		
rs717620 (*ABCC2*)	HWE *p* = 0.079	HWE *p* = 0.027		
Genotype				
AA	12	21	0.111	0.559 (0.271–1.153)
AG	121	100	0.092	1.308 (0.957–1.786)
GG	261	274	0.348	0.867 (0.643–1.169)
Allele				
A	145	142	0.826	1.029 (0.797–1.329)
G	643	648		
rs2273697 (*ABCC2*)	HWE *p* = 0.066	HWE *p* = 0.011		
Genotype				
AA	1	0	0.316	‐
AG	64	71	0.519	0.885 (0.611–1.283)
GG	329	324	0.583	1.109 (0.766–1.605)
Allele				
A	65	71	0.607	0.912 (0.641–1.296)
G	722	719		

a When calculating odds ratio of genotype AA, genotype AB together with genotype BB of this SNP were used as reference, vice versa.

b Chi‐square test for deviation from the Hardy–Weinberg equilibrium (a value of *p* < 0.001 was regarded as a deviation from the HWE).

### 3.3 Correlation between biochemical characteristics of HCV patients and genotypes of SNPs in STAT3 signaling pathway or HCV viral load

As listed in Table 3, genotypes of two SNPs in the STAT3 signaling pathway were associated with biochemical characteristics of HCV‐infected individuals. The HDL‐C level was significantly higher in patients with genotype AG than those with GG of rs4845617 in the *IL6* gene (*p* = 0.012). The GGT level were associated with genotypes of rs1053023 in the *STAT3* gene, and its level was lower in HCV‐infected persons with genotype AA than those with genotype AG (*p* = 0.011) (Table 3). Only three samples owned genotype AG of rs41279096, so we did not analyze the relationship between this SNP and biochemical characteristics of HCV patients.

**Table 3:** Association between biochemical characteristics and SNPs of genes in *STAT3* signaling pathway in HCV‐infected persons

Marker	rs1524107 (*IL6*)				rs2069837 (*IL6*)			
	CC	CT	TT	*P*‐value	AA	AG	GG	*P*‐value
GGT	46.92 ± 6.22	61.77 ± 6.14	54.28 ± 4.11	0.410	56.81 ± 4.07	58.35 ± 6.06	36.38 ± 9.21	0.512
ALT	70.85 ± 13.48	53.98 ± 3.79	75.89 ± 16.23	0.525	74.53 ± 13.99	54.75 ± 4.45	46.46 ± 11.58	0.559
AST	63.69 ± 9.24	54.56 ± 2.85	75.58 ± 17.84	0.696	73.74 ± 15.34	55.07 ± 3.31	45.69 ± 6.31	0.639
ALB	45.39 ± 0.81	44.37 ± 0.44	45.22 ± 0.24	0.156	45.04 ± 0.25	44.57 ± 0.45	45.74 ± 1.36	0.484
TBIL	14.10 ± 1.05	26.94 ± 6.79	15.04 ± 0.44	0.080	15.16 ± 0.43	28.68 ± 8.02	14.93 ± 1.55	0.051
HDL‐C	1.19 ± 0.08	1.24 ± 0.04	1.26 ± 0.03	0.770	1.25 ± 0.03	1.26 ± 0.04	1.16 ± 0.12	0.758
HCV RNA	4.90 ± 0.23	4.79 ± 0.10	4.76 ± 0.08	0.846	4.81 ± 0.08	4.74 ± 0.10	4.28 ± 0.51	0.461

Marker	rs2069852 (*IL6*)				rs4845617 (*IL6R*)			
	AA	AG	GG	*P*‐value	AA	AG	GG	*P*‐value
GGT	56.06 ± 4.74	57.02 ± 5.23	57.48 ± 9.47	0.986	57.69 ± 8.07	57.77 ± 4.65	54.22 ± 5.51	0.884
ALT	80.63 ± 19.15	52.95 ± 3.56	63.85 ± 8.79	0.368	58.93 ± 4.72	77.79 ± 19.42	57.77 ± 5.24	0.570
AST	81.91 ± 21.09	51.87 ± 2.53	58.98 ± 6.10	0.358	57.75 ± 3.33	76.87 ± 21.40	58.58 ± 4.09	0.650
ALB	45.30 ± 0.26	44.65 ± 0.38	44.30 ± 0.75	0.232	44.92 ± 0.46	44.66 ± 0.33	45.28 ± 0.38	0.472
TBIL	15.19 ± 0.48	24.43 ± 6.08	19.39 ± 5.67	0.250	16.79 ± 0.82	21.29 ± 4.63	18.70 ± 4.59	0.783
HDL‐C	1.28 ± 0.04	1.25 ± 0.04	1.18 ± 0.06	0.416	1.28 ± 0.05	1.30 ± 0.04	1.17 ± 0.04	0.045
HCV	4.77 ± 0.09	4.83 ± 0.10	4.63 ± 0.18	0.588	4.71 ± 0.11	4.90 ± 0.10	4.66 ± 0.11	0.208

Marker	rs4075015 (*IL6R*)				rs7553796 (*IL6R*)			
	AA	AT	TT	*P*‐value	AA	AC	CC	*P*‐value
GGT	58.70 ± 7.10	53.80 ± 4.48	58.49 ± 5.99	0.777	44.97 ± 7.11	56.41 ± 4.19	62.45 ± 6.53	0.200
ALT	57.77 ± 6.26	58.12 ± 4.32	82.04 ± 23.12	0.445	46.11 ± 4.90	62.07 ± 4.32	84.40 ± 25.23	0.322
AST	59.70 ± 5.50	53.31 ± 2.52	84.94 ± 25.58	0.348	48.70 ± 3.38	59.15 ± 3.24	85.92 ± 27.92	0.347
ALB	45.01 ± 0.50	45.19 ± 0.33	44.56 ± 0.37	0.435	45.06 ± 0.51	45.05 ± 0.33	44.65 ± 0.36	0.681
TBIL	14.30 ± 0.84	18.98 ± 3.63	22.79 ± 5.51	0.480	22.12 ± 8.66	19.66 ± 4.24	17.97 ± 2.03	0.863
HDL‐C	1.18 ± 0.05	1.25 ± 0.04	1.29 ± 0.04	0.266	1.17 ± 0.05	1.25 ± 0.03	1.30 ± 0.05	0.215
HCV	4.65 ± 0.12	4.90 ± 0.10	4.73 ± 0.10	0.259	4.66 ± 0.15	4.85 ± 0.09	4.72 ± 0.10	0.456

Marker	rs1053023 (*STAT3*)				rs1053004 (*STAT3*)			
	AA	AG	GG	*P*‐value	CC	CT	TT	*P*‐value
GGT	47.09 ± 3.89	64.14 ± 5.36	64.88 ± 13.53	0.034	64.81 ± 12.61	61.69 ± 5.15	48.90 ± 4.16	0.132
ALT	54.16 ± 3.17	61.68 ± 4.00	63.71 ± 11.63	0.345	59.64 ± 10.28	60.63 ± 4.03	55.75 ± 3.73	0.683
AST	57.38 ± 3.42	56.52 ± 2.40	58.39 ± 7.54	0.960	56.94 ± 6.69	57.89 ± 2.86	56.10 ± 3.06	0.913
ALB	45.31 ± 0.29	44.64 ± 0.36	44.34 ± 0.76	0.259	44.22 ± 0.67	44.66 ± 0.36	45.36 ± 0.28	0.190
TBIL	19.18 ± 4.55	17.14 ± 1.59	34.74 ± 17.80	0.201	31.60 ± 15.42	17.11 ± 1.54	19.51 ± 4.83	0.293
HDL‐C	1.28 ± 0.03	1.24 ± 0.04	1.19 ± 0.11	0.581	1.18 ± 0.09	1.26 ± 0.04	1.26 ± 0.04	0.597
HCV	4.79 ± 0.10	4.74 ± 0.09	4.93 ± 0.20	0.703	4.90 ± 0.20	4.76 ± 0.09	4.77 ± 0.10	0.821

Marker	rs4796793 (*STAT3*)				rs3787349 (*HNF4A*)			
	CC	CG	GG	*P*‐value	AA	AC	CC	*P*‐value
GGT	48.86 ± 4.00	59.78 ± 5.25	71.28 ± 11.45	0.087	34.25 ± 5.98	54.53 ± 4.71	59.60 ± 4.73	0.286
ALT	55.58 ± 3.97	58.87 ± 3.91	67.79 ± 9.20	0.414	49.25 ± 9.07	81.99 ± 23.29	58.76 ± 3.67	0.442
AST	57.66 ± 3.67	55.01 ± 2.36	64.05 ± 6.11	0.393	52.31 ± 6.71	84.27 ± 25.79	56.27 ± 2.45	0.389
ALB	45.25 ± 0.29	44.81 ± 0.34	44.11 ± 0.76	0.287	45.12 ± 0.60	44.86 ± 0.38	44.93 ± 0.28	0.968
TBIL	14.54 ± 0.47	20.78 ± 4.37	31.88 ± 13.37	0.130	14.23 ± 1.04	15.74 ± 0.68	14.75 ± 0.43	0.372
HDL‐C	1.30 ± 0.04	1.21 ± 0.03	1.27 ± 0.09	0.246	1.26 ± 0.15	1.23 ± 0.04	1.27 ± 0.03	0.829
HCV	4.82 ± 0.10	4.74 ± 0.09	4.81 ± 0.19	0.817	4.99 ± 0.35	4.89 ± 0.10	4.68 ± 0.08	0.204

Marker	rs3212172 (*HNF4A*)				rs1800963 (*HNF4A*)			
	AA	AG	GG	*P*‐value	AA	AC	CC	*P*‐value
GGT	58.81 ± 4.83	52.47 ± 4.52	63.90 ± 14.39	0.540	44.57 ± 7.82	59.40 ± 4.82	55.74 ± 5.08	0.498
ALT	57.40 ± 3.31	78.82 ± 22.19	74.10 ± 14.94	0.523	51.77 ± 8.36	77.88 ± 18.93	58.70 ± 3.98	0.539
AST	58.80 ± 2.79	77.58 ± 24.47	65.10 ± 8.45	0.669	48.93 ± 4.32	78.41 ± 20.85	57.66 ± 3.01	0.534
ALB	44.99 ± 0.25	44.88 ± 0.40	44.50 ± 0.93	0.846	45.01 ± 0.92	44.91 ± 0.31	44.90 ± 0.33	0.992
TBIL	21.43 ± 4.74	17.73 ± 1.91	15.19 ± 1.11	0.712	14.11 ± 1.00	20.24 ± 4.27	19.58 ± 3.61	0.833
HDL‐C	1.29 ± 0.03	1.22 ± 0.04	1.18 ± 0.07	0.325	1.21 ± 0.08	1.26 ± 0.04	1.25 ± 0.04	0.866
HCV	4.71 ± 0.08	4.90 ± 0.10	4.64 ± 0.23	0.279	4.52 ± 0.27	4.85 ± 0.09	4.74 ± 0.09	0.387

Marker	rs6073435 (*HNF4A*)				rs1169309 (*HNF1A*)			
	AA	AT	TT	*P*‐value	GG	GT	TT	*P*‐value
GGT	56.17 ± 4.92	58.03 ± 5.07	51.51 ± 7.61	0.845	51.21 ± 4.59	64.35 ± 5.47	46.12 ± 5.51	0.052
ALT	80.98 ± 21.92	58.41 ± 3.76	54.77 ± 8.07	0.461	62.81 ± 6.05	76.69 ± 17.86	52.07 ± 4.63	0.541
AST	80.50 ± 24.14	58.16 ± 3.09	53.67 ± 5.39	0.522	58.67 ± 3.95	77.88 ± 19.70	52.33 ± 3.48	0.528
ALB	45.14 ± 0.29	44.80 ± 0.35	44.49 ± 0.74	0.631	44.66 ± 0.49	44.89 ± 0.31	45.25 ± 0.39	0.634
TBIL	23.71 ± 6.06	16.74 ± 1.48	15.61 ± 2.49	0.396	21.57 ± 5.51	16.40 ± 1.46	23.72 ± 8.67	0.473
HDL‐C	1.26 ± 0.04	1.23 ± 0.03	1.33 ± 0.09	0.523	1.24 ± 0.05	1.26 ± 0.03	1.25 ± 0.05	0.945
HCV	4.82 ± 0.09	4.78 ± 0.09	4.54 ± 0.22	0.501	4.73 ± 0.11	4.81 ± 0.09	4.76 ± 0.13	0.878

Marker	rs11065390 (*HNF1A*)				rs1885301 (*ABCC2*)			
	AA	AG	GG	*P*‐value	AA	AG	GG	*P*‐value
GGT	47.00 ± 6.55	60.44 ± 5.44	54.89 ± 4.53	0.533	69.54 ± 17.59	62.34 ± 6.03	52.92 ± 3.99	0.313
ALT	45.65 ± 6.27	63.64 ± 4.58	72.77 ± 17.02	0.735	82.31 ± 21.61	91.36 ± 26.62	53.72 ± 3.13	0.152
AST	51.00 ± 5.67	59.35 ± 3.04	74.59 ± 18.78	0.702	64.54 ± 9.45	90.33 ± 29.41	54.51 ± 2.48	0.245
ALB	43.80 ± 0.92	44.88 ± 0.34	45.08 ± 0.30	0.374	47.94 ± 0.77	44.64 ± 0.35	44.90 ± 0.29	0.034
TBIL	36.51 ± 21.89	16.53 ± 1.76	19.62 ± 3.86	0.185	14.65 ± 1.44	25.79 ± 7.38	16.38 ± 1.21	0.224
HDL‐C	1.11 ± 0.08	1.27 ± 0.04	1.26 ± 0.03	0.312	1.23 ± 0.16	1.22 ± 0.04	1.27 ± 0.03	0.556
HCV	4.54 ± 0.31	4.93 ± 0.09	4.68 ± 0.08	0.091	4.48 ± 0.26	4.90 ± 0.10	4.73 ± 0.08	0.274

Marker	rs7910642 (*ABCC2*)				rs2804402 (*ABCC2*)			
	AA	AG	GG	*P*‐value	AA	AG	GG	*P*‐value
GGT	54.40 ± 9.42	57.93 ± 5.19	55.70 ± 4.64	0.926	52.80 ± 3.95	62.81 ± 6.16	69.54 ± 17.59	0.282
ALT	60.78 ± 8.95	56.92 ± 3.96	80.43 ± 21.09	0.468	54.12 ± 3.11	91.45 ± 27.23	82.31 ± 21.61	0.160
AST	58.43 ± 5.93	55.89 ± 3.12	81.12 ± 23.27	0.472	54.65 ± 2.46	90.90 ± 30.09	64.54 ± 9.45	0.241
ALB	45.44 ± 0.59	44.68 ± 0.32	45.04 ± 0.35	0.541	44.92 ± 0.29	44.58 ± 0.35	47.94 ± 0.77	0.030
TBIL	14.99 ± 0.76	19.29 ± 4.27	20.76 ± 3.83	0.816	19.54 ± 3.36	19.84 ± 4.46	14.65 ± 1.44	0.941
HDL‐C	1.32 ± 0.07	1.25 ± 0.03	1.25 ± 0.04	0.639	1.28 ± 0.03	1.21 ± 0.04	1.22 ± 0.16	0.495
HCV	4.63 ± 0.20	4.82 ± 0.09	4.76 ± 0.09	0.653	4.73 ± 0.08	4.91 ± 0.10	4.48 ± 0.26	0.239

Marker	rs717620 (*ABCC2*)			
	AA	AG	GG	*P*‐value
GGT	66.50 ± 18.84	64.18 ± 6.52	52.67 ± 3.83	0.240
ALT	83.33 ± 23.47	95.13 ± 29.01	53.62 ± 3.00	0.111
AST	65.58 ± 10.21	94.13 ± 32.07	54.25 ± 2.38	0.188
ALB	47.52 ± 0.70	44.62 ± 0.37	44.93 ± 0.28	0.090
TBIL	14.95 ± 1.53	20.16 ± 4.75	19.37 ± 3.24	0.944
HDL‐C	1.22 ± 0.18	1.21 ± 0.04	1.28 ± 0.03	0.458
HCV	4.42 ± 0.28	4.89 ± 0.11	4.74 ± 0.08	0.278

Marker	rs2069840 (*IL6*)			rs12090237 (*IL6R*)			rs4845374 (*IL6R*)		
	CC	CG	*P*‐value	AA/AG	GG	*P*‐value	AA/AT	TT	*P*‐value
GGT	56.03 ± 3.44	61.24 ± 10.94	0.614	57.84 ± 9.97	56.52 ± 3.47	0.914	62.07 ± 6.26	54.13 ± 3.84	0.262
ALT	67.44 ± 10.29	66.00 ± 9.57	0.960	174.0 ± 111.9	58.16 ± 2.81	0.0006	84.64 ± 28.25	59.30 ± 3.37	0.200
AST	67.45 ± 11.25	62.16 ± 6.56	0.867	183.9 ± 124.6	56.84 ± 2.07	0.0006	87.15 ± 31.33	57.52 ± 2.39	0.169
ALB	45.07 ± 0.23	43.70 ± 0.75	0.048	44.43 ± 0.92	44.95 ± 0.23	0.522	44.84 ± 0.38	44.95 ± 0.27	0.818
TBIL	20.15 ± 2.92	14.26 ± 1.23	0.470	18.41 ± 2.21	19.57 ± 2.81	0.905	24.02 ± 6.75	17.39 ± 2.17	0.235
HDL‐C	1.27 ± 0.03	1.16 ± 0.06	0.192	1.32 ± 0.11	1.25 ± 0.03	0.463	1.23 ± 0.04	1.27 ± 0.03	0.505
HCV	4.74 ± 0.06	5.02 ± 0.18	0.136	5.11 ± 0.24	4.75 ± 0.06	0.138	4.73 ± 0.12	4.80 ± 0.07	0.572

Marker	rs4509570 (*IL6R*)			rs2273697 (*ABCC2*)		
	CC	CG/GG	*P*‐value	AA/AG	GG	*P*‐value
GGT	58.57 ± 3.95	50.90 ± 5.72	0.310	55.40 ± 7.64	56.87 ± 3.64	0.868
ALT	71.42 ± 12.20	55.08 ± 4.52	0.439	107.6 ± 53.73	59.32 ± 2.91	0.051
AST	70.83 ± 13.34	55.11 ± 3.34	0.494	113.7 ± 59.64	57.59 ± 2.14	0.037
ALB	45.03 ± 0.24	44.56 ± 0.51	0.348	45.14 ± 0.49	44.87 ± 0.25	0.648
TBIL	18.63 ± 2.86	21.97 ± 5.80	0.576	16.99 ± 1.71	19.97 ± 3.09	0.670
HDL‐C	1.27 ± 0.03	1.20 ± 0.04	0.230	1.35 ± 0.07	1.24 ± 0.03	0.088
HCV	4.78 ± 0.07	4.78 ± 0.12	0.990	4.83 ± 0.15	4.77 ± 0.07	0.717

Note

All biochemical characteristic were Mean ± SE.

Abbreviations: ALB, albumin; ALT (or GPT), glutamic‐pyruvic transaminase; AST (or GOT), aspartate amino‐transferase; GGT, Glutamyl transpeptidase; HDL‐C, high‐density lipoprotein cholesterol; TBIL, total bilirubin

HCV RNA of a total of 266 HCV‐infected persons were extracted, but HCV genotypes were successfully determined in 105 patients due to long amplicon. The results showed that 16, 19, 5, 58, 1, and 6 patients infected by HCV with genotype 1B, 2A, 3A, 3B, 6A, and 6N, respectively. Genotype 3B was the frequent genotype (58/105, 55.24%) in this study, and this distribution was similar with the reported study in Yunnan (Li et al., 2017). HCV viral load was quantified in all 266 HCV‐infected persons. Because only one individual belonged genotype 6A, this genotype was excluded in further biochemical characteristics analysis. The ALT (*p* = 0.002), AST (*p* = 0.0001), ALB level (*p* = 0.047), and HCV viral load (*p* = 0.011) showed significant difference among HCV subgroups (Table 4). ALT (83.53 ± 7.34) and AST (80.40 ± 6.56) expressed higher level in HCV‐infected persons with genotype 3B. ALB level (39.41 ± 1.85) and HCV viral load (5.19 ± 0.42) was much lower in HCV‐infected persons with genotype 2A.

**Table 4:** Analysis of biochemical features and viral load among patients with various HCV genotypes

	1B (*n* = 16)	2A (*n* = 19)	3A (*n* = 5)	3B (*n* = 58)	6N (*n* = 6)	*P*‐value
GGT	77.06 ± 25.90	45.21 ± 15.23	87.20 ± 26.97	70.95 ± 7.89	47.00 ± 18.19	0.503
ALT	48.38 ± 10.69	29.39 ± 5.83	72.60 ± 22.69	83.53 ± 7.34	52.50 ± 29.23	0.002
AST	47.25 ± 5.50	34.78 ± 4.75	49.40 ± 4.37	80.40 ± 6.56	34.67 ± 9.17	0.0001
ALB	44.00 ± 1.12	39.41 ± 1.85	42.78 ± 3.37	43.76 ± 0.59	43.65 ± 1.28	0.047
TBIL	12.66 ± 1.58	21.80 ± 5.92	14.98 ± 4.05	16.22 ± 1.01	13.22 ± 2.04	0.283
HDL‐C	1.25 ± 0.08	0.96 ± 0.13	1.08 ± 0.10	1.19 ± 0.06	0.96 ± 0.14	0.194
Viral load	5.89 ± 0.17	5.19 ± 0.42	5.63 ± 0.28	5.33 ± 0.07	6.16 ± 0.40	0.011

Abbreviations: ALB, albumin; ALT (or GPT), glutamic‐pyruvic transaminase; AST (or GOT), aspartate amino‐transferase; GGT, Glutamyl transpeptidase; HDL‐C, high‐density lipoprotein cholesterol; TBIL, total bilirubin.

### 3.4 Variation of mRNA and protein expression level after HCV infection with or without IL6 transfection

**Figure 1:**
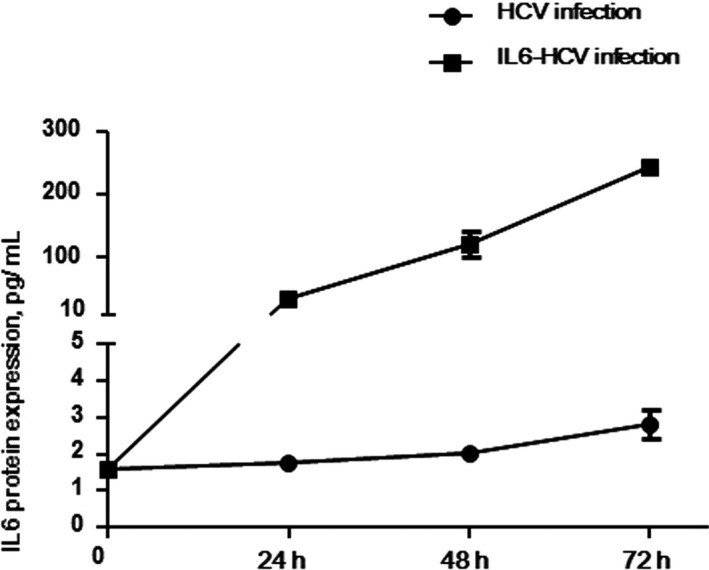
IL6 expression level in HCV infected cells with or without IL6 transfection. The IL6 protein level was detected in cells infected by HCV and cells transfected with the *IL6* gene and infected by HCV

**Figure 2:**
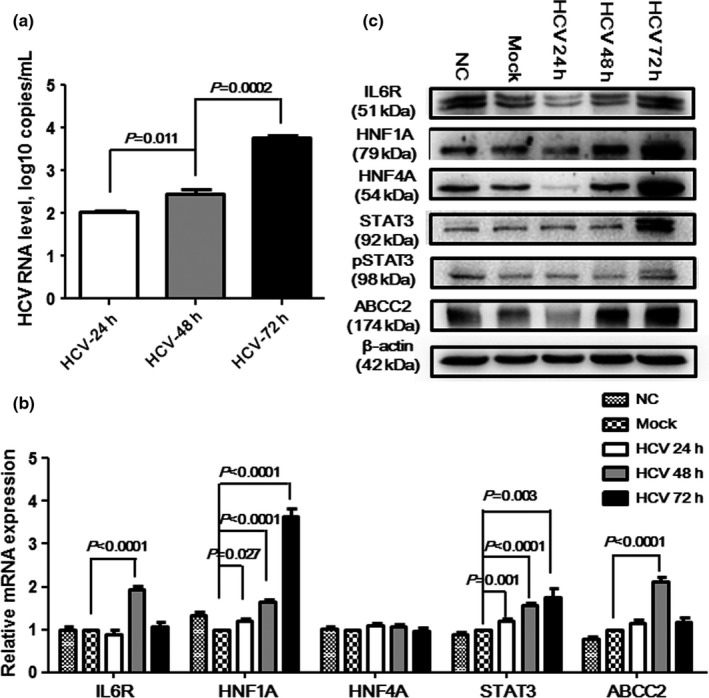
HCV viral load and expression level of genes in STAT3 signaling pathway after HCV infection. (a) time‐dependent increasing HCV RNA level; (b) mRNA expression level variation of the IL6R, HNF1A, HNF4A, STAT3, and ABCC2 genes at 24, 48, and 72 hr after the Huh7.5.1 cells were infected with HCV; (c) expression level of the IL6R, HNF1A, HNF4A, STAT3, pSTAT3, and ABCC2 proteins at 24, 48, and 72 hr after the Huh7.5.1 cells were infected with HCV. NC means normal controls, i.e., the cells without HCV infection; mock means the negative controls, i.e., the cells were infected with PBS

All above results suggested genetic polymorphisms in the STAT3 signaling pathway might be associated with HCV infection, so functional assays were further performed to identify the role of this pathway. We studied mRNA and protein expression of six genes in STAT3 signaling pathway, after Huh7.5.1 cells were infected by HCV with or without transfection of the *IL6* gene. We identified that IL6 protein expression gradually increased in HCV‐infected cells (Figure 1), but no statistical difference was found. HCV viral load time‐dependently increased from 24 to 72 hr after HCV infection (Figure 2a). The mRNA level of the *HNF1A* and *STAT3* genes showed time‐dependent increasing in HCV‐infected cells (Figure 2b). While mRNA level of the *IL6R* and *ABCC2* genes were highest in 48 hr (Figure 2b). The *HNF4A* gene showed no statistical difference in mRNA level. However, mRNA expression was different from proteins. All six proteins (IL6R, HNF4A, HNF4A, STAT3, pSTAT3, and MRP) in this pathway seemed high expression at 72 hr after HCV infection (Figure 2c).

**Figure 3:**
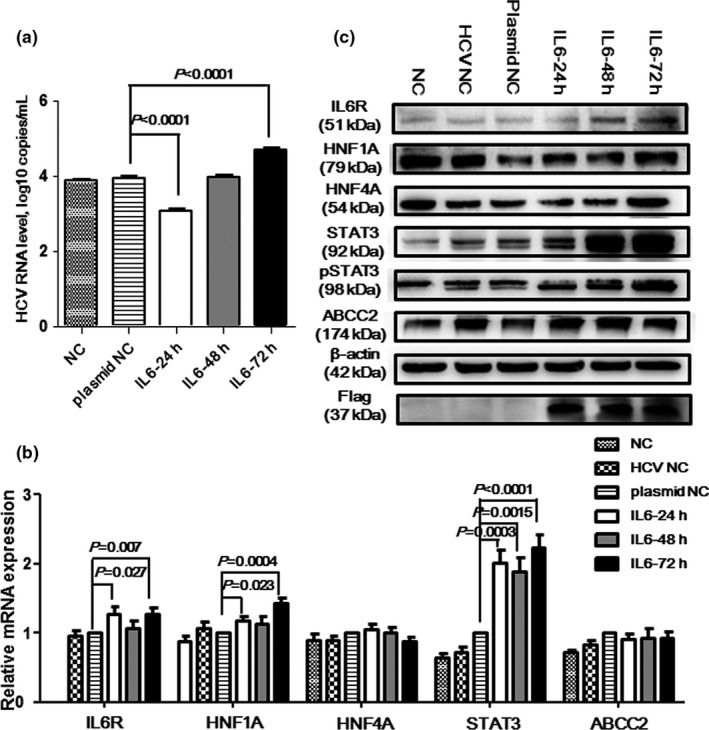
HCV viral load and expression level of genes in STAT3 signaling pathway after IL6 transfection and HCV infection. (a) HCV RNA level, the RNA level decreased at 24 hr after HCV infection in Huh7.5.1 cells transfected with IL6 gene; (b) mRNA expressing level variation of the IL6R, HNF1A, HNF4A, STAT3, and ABCC2 genes at 24, 48, and 72 hr after the HCV infection in Huh7.5.1 cells transfected with IL6 gene; (c) expressing level of the IL6R, HNF1A, HNF4A, STAT3, pSTAT3, and ABCC2 proteins at 24, 48, and 72 hr after HCV infection in Huh7.5.1 cells transfected with IL6 gene. NC means normal controls, i.e., the cells without HCV infection; HCV NC means cells infected by HCV; plasmid NC means cells transfected with the IL6 gene and infected by HCV

In order to detect whether STAT3 signaling pathway could influence HCV proliferation in vitro, the IL6 gene was overexpressed in Huh7.5.1 cells. After analyzing RNA and protein expression in IL6‐transfected and HCV‐infected cells, IL6 protein level significantly increased (Figure 1). The *P* value was 0.0001, 0.002, and 0.0008, respectively, when comparing expressing level at 24 hr with control, expressing level at 48 hr with that at 24 hr, and expressing level at 48 hr with that at 72 hr. Meanwhile, the expression of flag suggested that the IL6 gene was successfully transfected into cells. HCV viral load significantly decreased at 24 hr (*p* < 0.0001) and increased at 72 hr (*p* < 0.0001) after infection (Figure 3a). Similar to results of HCV infection, the mRNA level of the *IL6R*, *HNF1A*, and *STAT3* genes were significantly increased, but the mRNA level did not change in the *HNF4A* and *ABCC2* genes (Figure 3b). When we analyzed the proteins expression of the STAT3 signaling pathway, IL6R, HNF1A, HNF4A, 2STAT3, pSTAT3, and ABCC2 proteins time‐dependently increased (Figure 3c). These results identified that the transfected IL6 gene could effectively inhibit HCV proliferation by upregulating proteins expression level in STAT3 signaling pathway.

## 4 DISCUSSION

HCV infection was one of the seriously infectious hepatitis disease, so to further study and understand the mechanisms of HCV‐infection was needed. In this study, we collected several biochemical features of HCV‐infected persons to investigate whether there was the relationship between genetic polymorphisms in STAT3 signaling pathway and biochemical features. GGT, ALT, and AST levels in HCV patients were significantly higher than those in controls, and HDL‐C level in HCV patients was lower than that in controls (Table1). However, AST and HDL‐C levels were similar in male and female cohorts, respectively. These results indicated that different results of AST and HDL‐C level might be caused by gender difference, so more samples should be further studied. In our previous study, ALT/AST ratio and TBIL level were associated with SNPs in the *IFNλ* genes (Zhang et al., 2014), and GGT, AST, and TBIL levels were related with SNPs in the *NPC1L1* gene of the HCV‐infected persons (Zhang et al., 2016). In addition, SNPs in the *CD81*, SCARB1, and *CLDN1* genes, which were essential for HCV particle entry, were found to correlate with metabolic features of HCV patients (Nafari et al., 2018; Sun, Jin, & Kang, 2015). Similar to this study, ALT, AST, and HDL‐C level could be influenced by genotypes of SNP in these genes (Nafari et al., 2018; Sun et al., 2015). Together with our and others previous results, we suggested that biochemical features of HCV‐infected persons were associated with genetic polymorphisms of patients.

The proteins in STAT3 signaling pathway played important roles in HCV infection or HCC (Ji et al., 2018; Kao et al., 2017; Qadri et al., 2006). HCV infection could lead to increasing of HNF1A and HNF4A proteins, which caused upregulation of ABCC2 (Qadri et al., 2006). Aboulnasr *et al.* identified IFNλ could inhibit HCV replication through suppression miR122‐ STAT3‐ HNF4A feedback loop (Aboulnasr et al., 2015). However, in Vallianou's study, HCV infection might lead to HCC through decreasing of HNF4A, HNF1A, and ABCC2 (Vallianou, Dafou, Vassilaki, Mavromara, & Hadzopoulou‐Cladaras, 2016). This result differed from the study by Qadri *et al*. The miR‐125b has been reported to be an inhibitor for HCC (Kim et al., 2013), while IL6/STAT3 pathway could upregulate the expression of miR‐125b in HCV infected cells (Dai et al., 2018), which means that STAT3 pathway could prevent HCV infected cells from HCC. Although the results existed discrepancy in these studies, it suggested HCV infection and progression closely related with IL6/STAT3 signaling pathway. However, all these studies focused on the functional role of STAT3 pathway, and whether genetic SNPs of STAT3 pathway was associated with HCV infection was unknown. Thus, the precise role of STAT3 pathway needs further study.

Many host genetic factors have been studied to explore the susceptibility of HCV infection in different populations (Steba et al., 2018; Swiatek‐Koscielna et al., 2017; Zhang et al., 2014, 2016), but it seemed that no high coincident result in these cohorts. IL6 was the most reported gene to be associated with HCV infected persons or HCC patients caused by chronic HCV infection. SNP rs1800795 was associated with treatment of HCV patients infected by drug injection in Croatian (Bogdanovic et al., 2016). Moreover, the polymorphisms of the *IL6* gene were reported to influence the histologic progression and clinical outcomes of HCV patients (Falleti et al., 2010; Sghaier et al., 2017). Although daclatasvir plasma concentration significantly increased in the HCV infected patients with genotype CC of SNP rs1884613 in the *HNF4A* gene (Cusato et al., 2017), the relationship between SNPs in this gene and HCV infection was not studied. The genetic role of other four genes in STAT3 signaling pathway was not reported. We firstly identified that SNPs in the STAT3 signaling pathway were associated with HCV infection (*IL6R* and *HNF4A* genes) or biochemical features of HCV patients (*IL6* and *STAT3* genes) in Yunnan population. Thus, our results suggested that the genetic polymorphisms of the genes in STAT3 signaling pathway exerted important roles in HCV infection, biochemical features of patients, and even disease progression.

The treatment effect was different in patients with various HCV genotypes (Atsukawa et al., 2018; Di Biagio, Taramasso, & Cenderello, 2018; Otsuka et al., 2017). Similarly, we identified that the ALT, AST, ALB, and viral load level showed significant difference among the HCV patients with subtypes (Table 4). This indicated that HCV genotypes might influence the biochemical features and treatment of HCV patients. High ALB level in the patients with HCV genotype 2A might be due to the lower HCV viral load in these patients.

High IL6 level linked with phosphorylated STAT3 level, poor liver function, variceal severity, and patients' mortality in cirrhotic patients caused by HBV‐ and HCV‐infection (Kao et al., 2017). Similarly, Nakagawa *et al.* identified that high serum level of IL6 was associated with liver‐related mortality in chronic HCV patients (Nakagawa et al., 2015). Moreover, plasma level of IL6 could be a predicted index of interferon‐α plus ribavirin therapy in HIV/HCV co‐infected patients (Guzman‐Fulgencio et al., 2012). In this study, we identified that HCV RNA level significantly increased at 24, 48, and 72 hr after HCV infection, but it significantly decreased after 24 hr transfection of the IL6 gene and HCV infection than in control cells. This meant that high expression of IL6 could dampen HCV reproduction (Figure 3a). Similar to Qadri's results (Qadri et al., 2006), we found that mRNA and proteins expression of IL6R, HNF1A, STAT3, and ABCC2 (ABCC2) significantly increased in cells infected by HCV with or without IL6 transfection, but HNF4A showed no statistical difference. Interestingly, HNF1A, HNF4A, and ABCC2 were downregulated by HCV infection, but IL6R and STAT3 increased in another research (Vallianou et al., 2016). It has been reported that downregulation of STAT3 could suppress hepatocytes proliferation and enhance the anti‐apoptotic ability of inflammatory hepatocytes (Starkel, Saeger, Leclercq, & Horsmans, 2007). Thus, we suggested that upregulated STAT3 might slow down the progression of hepatocytes caused by HCV infection. However, the function of the STAT3 signaling pathway in HCV infection needed further verification in animal model.

We firstly identified that genetic polymorphisms of the genes in STAT3 signaling pathway were associated with HCV infection and biochemical features of HCV patients in Yunnan population. In summary, the genes in the STAT3 signaling pathway played important roles in HCV infection through both genetic and functional aspects.

## CONFLICT OF INTEREST

The authors declare no conflict of interest.

## AUTHOR CONTRIBUTIONS

YZ Song and A‐M Zhang designed experiment; YZ Song, XY Yang, and YQ Wang performed experiments; YS Shen and XS Xia collected the patients; YZ Song and A‐M Zhang analyzed the data; A‐M Zhang prepared the manuscript. All authors have agreed to this submission. Our study has not been previously published.
