# Supplementary figures and images for: STAT3 signaling pathway plays importantly genetic and functional roles in HCV infection
Source: Mol Genet Genomic Med. 2019 Jun 20;7(8):e821. doi: 10.1002/mgg3.821 (PMC6687657; doi:10.1002/mgg3.821)

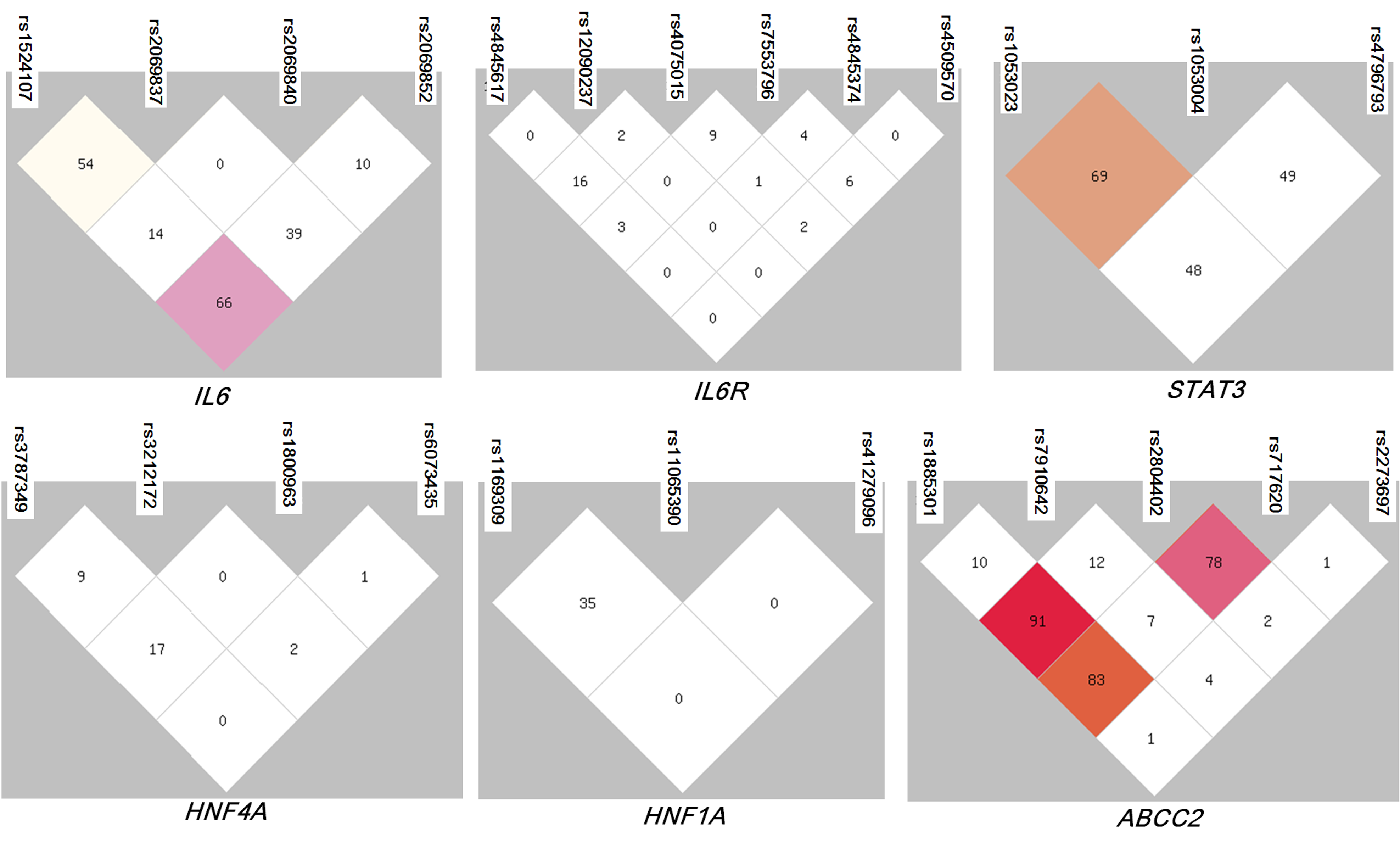

Supplement: Supplementary file 1 [file MGG3-7-e821-s001.tif]
