# Supplementary material for: STAT3 signaling pathway plays importantly genetic and functional roles in HCV infection
Source: Mol Genet Genomic Med. 2019 Jun 20;7(8):e821. doi: 10.1002/mgg3.821 (PMC6687657; doi:10.1002/mgg3.821)
Supplement: Supplementary file 2 [file MGG3-7-e821-s002.doc]

Table S1. Primers for SnapShot assay.

| SNP | Forward primers (5’→3’) | Reverse primers (5’→3’) | Extending primers (5’→3’) |
| --- | --- | --- | --- |
| rs1524107 | ACTCCACTGGAATTTGCTTGC | AGCCATCTTTGGAAGGTTCAG | TTTTTTGTTCAACCACAGCCAGGAAA |
| rs2069837 | TAAAATTGGGCGTGGACTAGG | TCACCTGTGACTTTAGGTGTG | GAAGATTAGACACAATATTTAT |
| rs2069840 | CAATGAGGTACCAACTTGTCG | AAAATCTCACCACAGTGGCAC | TTTTTTTTTTTTTTTTGTAAATTTCATGAGGAGGCCAA |
| rs2069852 | TAAGCACCTACTACATGCCAG | TACATCATCCCTCAGTTCCTG | TTTTTTTTTTTAAATTACTTAGTCTTCCACAA |
| rs4845617 | TCGGCCGGTGCGCGGGGCTGT | TCTCTACACACACTGCGAGTC | TTTTAGTCGCACTGACACTGAGCCGG |
| rs12090237 | AGAGATAGAAGACAAGACCCC | TGTGTAGGGTGCTGAGATAAG | CAGCAGTGCATGGTGTCAGGAC |
| rs4075015 | CCTGAGGATGCTTCTGTTCAGT | CATGTGTCTACCCAAGCTCAT | TTTTTTTTTTTTACTCTTGCATTCCAATCACTGC |
| rs7553796 | GCAGGAATGCTGAACAAAGTG | TATTGAAGTCACTGCTCTGGC | TTTTTTTTTTTTTTTTTTTTTTAAATATTAAATGGGAAATTCCA |
| rs4845374 | ATTTTCTGGGAGGGGGGTTGGA | CTCAGAACAATGGCAATGCAG | TTTTTTTTTTTTTTTTTTTTTCCTCTTCCTCCTCTATCTTCAA |
| rs4509570 | AGTTACTGAGCCCTGGCTTTG | CTTGCTCCTAATGTTGCTGAC | TTTTTTTTTTTCAAATTGTGCCCAGTGGTGCC |
| rs1053023 | CAAGTTCATGGCCTTAGGTAG | GACAGCAGCTTATAAACCACC | GAGCTGAGCCCTGTTGTGGCCC |
| rs3787349 | AGCTAAGCAGGGCAGAGAAA | TGACACCTGTGAGCAAGAGTGG | TTTTTTTTTTACTCACTCATTAATTCACCCAC |
| rs3212172 | AGCTAAGCAGGGCAGAGAAA | TGACACCTGTGAGCAAGAGTGG | TTTTTTTTTTTTTTTTTTTCATTCACTCACTCATTCATGC |
| rs41279096 | AGGGACAAAGGAGCCTGTGAAC | TTTCCACCGCATTTCTCCTTG | AAAGTTAAATAAACGTGCCACT |
| rs7910642 | TTTAGCTAGGATACCGCATGGG | AATGTTTTCTGTAGGGACGGG | CCCAGCCCTTTGGGAGGCCAAG |
| rs2804402 | TTTAGCTAGGATACCGCATGGG | AATGTTTTCTGTAGGGACGGG | AACTCCAGGCTTCAACAATCCT |
