# Supplementary material for: STAT3 signaling pathway plays importantly genetic and functional roles in HCV infection
Source: Mol Genet Genomic Med. 2019 Jun 20;7(8):e821. doi: 10.1002/mgg3.821 (PMC6687657; doi:10.1002/mgg3.821)
Supplement: Supplementary file 3 [file MGG3-7-e821-s003.doc]

Table S2. Primers and probes for Ligase Detection Reaction.

| SNP | Forward primers (5’→3’) | Reverse primers (5’→3’) |
| --- | --- | --- |
| rs1053004 | TCGTATGAGGGTGTATACCTG | GGTTTTGAGTTGCCAAATCCG |
| rs4796793 | GCTGTTTATCTACACCACAGG | AATATGGCCTCTCCTATCTGC |
| rs1800963 | GCCTGAATGTAAATCCAGGTC | AGAATGGACTGGAAGTTTGGG |
| rs6073435 | GTTTGAGAAAGAAGGGCAAGG | GGATGATTGAGGGAGACTTTG |
| rs1169309 | CTCCCCACTCTGCTCTGATG | CAGAGTGGTGTAGCGGTCC |
| rs11065390 | CTCCTTCCAGCTAGTGACCC | CTTCCCAGTAGAGGCAGGAG |
| rs2273697 | TCCATATGGAGCACATCCTTC | GAATGGTCTTACTCTTGGTGG |
| rs1885301 | CAGTTGTCATATACCTGTTGGC | GAAAAGGCAGCATTCAGTGTG |
| rs717620 | CTACCACTTGTTCTGAGTCTG | CGGAGAACATCAGAATGGTAG |

| SNP | Probe 1 | Probe 2 | Probe 3 |
| --- | --- | --- | --- |
| rs1053004 | CCTGCCCGCCTTGGCTGGCTAGCTC | TTTCCTGCCCGCCTTGGCTGGCTAGCTT | GCCTCTCCTGTGCGTATGGGAACAC |
| rs4796793 | TTTAGTGATTTACTGCTTACAAAGC | TTTTTTAGTGATTTACTGCTTACAAAGG | ACTTACACACCTTATACACTCCCTG |
| rs1800963 | TTTTAACACTGGGAGCTGTGGGAGACGGA | TTTTTTTAACACTGGGAGCTGTGGGAGACGGC | GAGGGGCAGGGTGGGATCACAGGGATTT |
| rs6073435 | TTCAGAAATTTGGATTTTATTCTCA | TTTTTCAGAAATTTGGATTTTATTCTCT | TTGCAACTATTGTAAGGAGAGTGAA |
| rs1169309 | TTTTTTTTTTTTCCCAACCCGTGGAGGCTGCTCGGGG | TTTTTTTTTTTTTTTCCCAACCCGTGGAGGCTGCTCGGGT | TGCACAGGAGGGGGTCGTGGAGAGCTTTTTTTTT |
| rs11065390 | TTTTTTTTTTTTTTTTTGTGCCAGAGCCTGGGGCTCTAACA | TTTTTTTTTTTTTTTTTTTTGTGCCAGAGCCTGGGGCTCTAACG | CCTGAGCCCAGGGAGGCCGAAGCTATTTTTTTTTTTT |
| rs2273697 | TTTTTTTTTTTTAACTTGGCCAGGAAGGAGTACACCA | TTTTTTTTTTTTTTTAACTTGGCCAGGAAGGAGTACACCG | TTGGAGAAACAGTGAACCTGATGTCTTTTTTTTT |
| rs1885301 | TTTTAGTTGTATGAGTTCCTTATAGTATA | TTTTTTTAGTTGTATGAGTTCCTTATAGTATG | TTGTGGATATTAACTCTTCATCAGTTTT |
| rs717620 | TTTTTTTTTGATTCCTGGACTGCGTCTGGAACA | TTTTTTTTTTTTGATTCCTGGACTGCGTCTGGAACG | AAGACTCTTCTATTAATATGATTGTTTTTTT |
