# Supplementary material for: STAT3 signaling pathway plays importantly genetic and functional roles in HCV infection
Source: Mol Genet Genomic Med. 2019 Jun 20;7(8):e821. doi: 10.1002/mgg3.821 (PMC6687657; doi:10.1002/mgg3.821)
Supplement: Supplementary file 4 [file MGG3-7-e821-s004.doc]

Table S3. The primers for real-time PCR.

| Gene | Primer | Sequence (5’→3’) | GenBank Reference Sequence Number |
| --- | --- | --- | --- |
| *IL6R* | IL6R-F | TGCCAGGTGACACTGAGCC | NM_000600 |
|  | IL6R-R | TCGCCAGTAGTGTCGGGAGC |  |
| *HNF4A* | HNF4A-F | CGTGCTGCTCCTAGGCAA | NM_001258355 |
|  | HNF4A-R | GTCAAGGATGCGTATGGACAC |  |
| *HNF1A* | HNF1A-F | CCATGACCTCCAGCTTTCC | NM_001306179 |
|  | HNF1A-R | GTAAGGACGACTTCCCAGC |  |
| *STAT3* | STAT3-F | CCTCCTCCTTGGGAATGTC | NM_139276 |
|  | STAT3-R | AAGGACATCAGCGGTAAGAC |  |
| *ABCC2* | ABCC2-F | CAGGGCTCTGCTTCGGAAATC | NM_000392 |
|  | ABCC2-R | AACTCGTTTTGGATGGTCGTCTG |  |
| *GAPDH* | GAPDH-F | GGCATCCTGGGCTACACTGAG | NM_001256799 |
|  | GAPDH-R | CATACCAGGAAATGAGCTTGAC |  |
